# Evaluation of Neonatal Cerebral Circulation Under Hypoxic Ischemic Risk Factors Based on Quantitative Analysis of Cerebral Veins with Magnetic Resonance Susceptibility Weighted Imaging

**DOI:** 10.1007/s00062-024-01432-0

**Published:** 2024-06-26

**Authors:** Qi Xie, Yan-Hui Liao, Wen-juan He, Peng-peng Han, Jun Wu

**Affiliations:** 1 Medical Imaging Department of Nansha, Guangzhou First People’s Hospital, School of Medicine, South China University of Technology, 511457 Guangzhou, China; 2 Institute of Software Application Technology, 511458 Guangzhou, China; 3 Department of Nuclear Medicine, Meizhou, People’s Hospital, 514031 Meizhou, China

**Keywords:** MRI, SWI, Neonatal Hypoxic-Ischemia, DMVs, Image Segmentation Algorithm

## Abstract

**Purpose:**

To observe the regulation of cerebral circulation in vivo based on image segmentation algorithms for deep learning in medical imaging to automatically detect and quantify the neonatal deep medullary veins (DMVs) on susceptibility weighted imaging (SWI) images. To evaluate early cerebral circulation self-rescue for neonates undergoing risk of cerebral hypoxia-ischaemia in vivo.

**Methods:**

SWI images and clinical data of 317 neonates with or without risk of cerebral hypoxia-ischaemia were analyzed. Quantitative parameters showing the number, width, and curvature of DMVs were obtained using an image segmentation algorithm.

**Results:**

The number of DMVs was greater in males than in females (*p* < 0.01), and in term than in preterm infants (*p* = 0.001). The width of DMVs was greater in term than in preterm infants (*p* < 0.01), in low-risk than in high-risk group (*p* < 0.01), and in neonates without intracranial extracerebral haemorrhage (ICECH) than with ICECH (*p* < 0.05). The curvature of DMVs was greater in term than in preterm infants (*P* < 0.05). The width of both bilateral thalamic veins and anterior caudate nucleus veins were positively correlated with the number of DMVs; the width of bilateral thalamic veins was positively correlated with the width of DMVs.

**Conclusion:**

The DMVs quantification based on image segmentation algorithm may provide more detailed and stable quantitative information in neonate. SWI vein quantification may be an observable indicator for in vivo assessment of cerebral circulation self-regulation in neonatal hypoxic-ischemic brain injury.

## Introduction

The perinatal fetus or neonate is at risk for hypoxic-ischemic brain injury when experiencing factors that can reduce oxygen exchange and circulation function [1]. These children are in a special developmental period that their brain tissue and cerebrovascular development are not perfected [1–4], and more susceptible to white matter injury (WMI) due to hypoxia-ischemia altering specific trajectories in neuronal and glial cell development [1, 5–7]. When these children have clinical symptoms and signs, their brain injury have been moderate to severe, with a high disability rate and even death. The survivors have brain injury with neurological dysfunction [1].

WMI is an injury of neurovascular units involving oligodendrocytes, axons and vascular endothelial cells [8]. Previous studies have focused on oligodendrocytes and axons, with less research on vascular factors [1–4]. Deep medullary vein (DMV) is a white matter vein that drains to the corresponding subventricular vein and finally connects to the deep venous system. DMV has a unique confluence area with the subventricular vein [5, 9–11]. Over the past decade, neuroimaging has continued to reveal characteristic patterns of DMV thrombosis and peripheral tissue infarction [12–14]. DMV-related disease should be suspected when early magnetic resonance imaging (MRI) suggests a parallel or radial distribution of lesions in deep white matter areas [8]. In preterm infants, the immature deep venous system is more prone to venous congestion and thrombosis [15, 16].

Magnetic susceptibility weighted imaging (SWI) is based on the T2* weighted gradient echo sequence, which can obtain both phase and intensity information and fuse them through post-processing to obtain SWI and minimum intensity projection (MinIP) maps. MinIP map can highlight the magnetic sensitivity gap between different tissues and is highly sensitive in the visualization of intracranial veins (especially small veins, such as DMV) [7, 17]. DMV drain deep white matter venous blood into the internal cerebral veins via the subventricular veins [5, 9–11]. Kitamura et al. proposed the evaluation of the convexity grade of the brain DMV based on the SWI sequence MinIP images and developed a convexity grade system for the DMV [18]. However, this evaluation system is still subjective.

The regulation of self-blood circulation in children undergoing hypoxic-ischemic risk factors is a dynamic process [12, 19]. Abnormal oxygenation and changes in blood circulation precede brain injury [19]. Early identification of erroneous brain oxygenation and/or perfusion patterns in children may be important for correcting interventions as promptly as possible to prevent or at least reduce damage to the developing brain [19]. Therefore, using SWI to observe changes in DMV and its drainage veins has potential clinical value for in vivo imaging evaluation of early circulatory self-rescue changes in pediatric patients. With the development of computer and artificial intelligence techniques, more detailed quantification of veins on SWI images has become possible. In this study, we attempted to use the big data model trained based on image segmentation algorithm to automatically identify the DMV on the SWI MinIP image by computer and provide quantitative data. Meanwhile, we manually measured the width of the drainage veins of bilateral DMV beside the lateral ventricles. By analyzing the relationship between asymptomatic intracranial haemorrhage and the changes of brain DMV, and the differences of the DMV under the influence of various factors in neonates, we explored the possibility of SWI to observe and evaluate the cerebral circulation regulation function of neonates undergoing hypoxic-ischemic risk factors in vivo.

## Methods

### Subjects

This study is a retrospective analysis of clinical and MR data, approved by the local institutional Ethics Committee (No. K‑2019-166-01). Medical data of 358 consecutive newborns who were hospitalized in the neonatal intensive care unit of our hospital from December 2017 to November 2020 and completed head MRI scans were collected. 41 children with one of the following conditions were excluded: (I) had mechanical injuries such as forceps and suction delivery via vaginal delivery; (II) were older than 28 days; (III) had clinical manifestations of intracranial haemorrhage confirmed by an experienced paediatrician; (IV) had vitamin K deficiency; (V) absence of the SWI sequence; (VI) quality of MRI images were poor; (VII) and congenital malformations of the brain revealed by MRI images. 317 children finally included in the study. 111(35.02%) were preterm (gestational age < 37 weeks) and 206(64.98%) were full-term (gestational age ≥ 37 weeks).

High risk group: Children with a history of diseases that affect blood oxygen exchange and circulatory function, such as intrauterine infection, history of birth asphyxia, premature birth, neonatal pneumonia, neonatal hyaline membrane disease, sepsis and congenital heart disease (atrial septal defect, ventricular septal defect, patent ductus arteriosus), etc. There were 249(78.55%) cases, 129(51.81%) males and 120(48.19%) females, with an age of 7.3 ± 4.6 days at the time of MR examination.

Low-risk group: Children who undergo MR examination solely for hyperbilirubinemia to exclude brain injury. 68(21.45%) cases were included, 38(55.88%) males and 30(44.12%) females. The age at the time of MR examination was 10.3 ± 4.8 days.

### MR Examination

Newborns who experienced high-risk factors that affect blood oxygen exchange and circulatory function should undergo MR examination in the stable condition. All newborns were in sleep, warmth and accompanied by their parents throughout the examination. Informed consent was obtained from parents before MR scans.

A Skyra 3.0T MRI scanner with a 20-channel phased-array coil (Siemens, Erlangen, Germany) was used. The data of T1 weighted imaging and 3D SWI of the entire brain were included in the analysis of this study. The scanning parameters were as follows.

- T1WI_dark-fluid: repetition time (TR) = 1800 ms, echo time (TE) = 8.6 ms, slice thickness = 4 mm, slice gap = 1 mm, flip angle = 150°. The field of view (FOV) = 180 mm × 152 mm, the resolution matrix = 256 × 151.
- SWI: TR = 27 ms, TE = 20 ms, slice thickness = 1.5 mm, slice gap = 0.3 mm, flip angle = 15°, FOV = 180 mm × 163 mm, matrix = 256 × 223. MinIP map reconstruction thickness = 12 mm.

### Data Collection of Images

#### Quantitative Data Collection of DMVs

Deep learning and automatic quantification methods of image segmentation algorithm for DMV were the same as our previous research [20]. It was completed by Guangzhou Institute of Software Application Technology. The steps for image data processing are outlined below.

##### Processing of Raw Data and Collection of Data Sets for SWI

MinIP axial images parallel to the body of corpus callosum were reconstructed using the raw data from SWI volumetric imaging with a layer thickness of 20 mm and a interlayer distance of 1 mm. These MinIP axial images were used as the image data for the model dataset.

MinIP image data from 150 newborns enrolled in this study were imported into a MicroDicom viewer (https://www.microdicom.com/) to observe the DMV of the bilateral corona radiata in the fixed window width of 240 and window level of 200. Six layers axial images in which the DMVs were best visualized were selected, including the corpus callosum horizontally and the upper three layers and lower two layers. Finally, 900 sets of images were obtained.

##### Dataset Preprocessing and Model Training

These selected DICOM format images were converted to JPG format ones and imported into the open-source software Labelme (https://github.com/wkentaro/labelme). All visible DMVs contours were manually outlined one by one by the neuroradiologist with more than 2 years’ work experience.

The experiment was performed on Ubuntu 20.04 system, and the central processing unit (CPU) was an Inter Core Xeon E2124G, RTX3090 graphics card (Santa Clara, CA, USA). PyTorch framework version 1.4 (Linux Foundation, San Francisco, CA, USA) and Python version 3.6 (Python Software Foundation, Wilmington, DE, USA) were used. The DeepLabV3+ network (Hasty, Berlin, Germany) was used to train the segmentation image of DMV. The main body of this network structure decoder is a deep convolutional neural network (DCNN) with atrous convolution, and its basic network structure, Resnet, was used to extract image features. This network structure decoder also has atrous spatial pyramid pooling (ASPP), which is mainly used to introduce multiscale information and a decoder module, to further fuse low-level features with high-level features and improve the accuracy of segmentation boundaries. 1000 iterations were used. A gradual decrease method was adopted for the learning rate to avoid falling into a local optimum; specifically, after every 200 iterations, the learning rate was adjusted. The input image size was fixed to 512 × 512, and the output data were the model classification prediction results.

The main parameters for training the deep learning model included: model depth = 19, hidden layer cfg = 16, dropout = 0.5, batch size = 64, pretrained model = true, number of training iterations (number of epochs) = 1000, and learning rate = 0.01.

##### Model Evaluation

4‑fold cross validation was performed on 900 sets of data. All data were randomly divided into training, validation, and testing sets in a 5:2:2 ratio. In the cross-validation experiment, the DeepLabV3 + network was used on four training and validation sets to obtain the average accuracy of the four experiments. According to the 4‑fold cross validation results, the average test set accuracy of the four experiments was 98.0325%.

##### Model Application

**Fig. 1:**
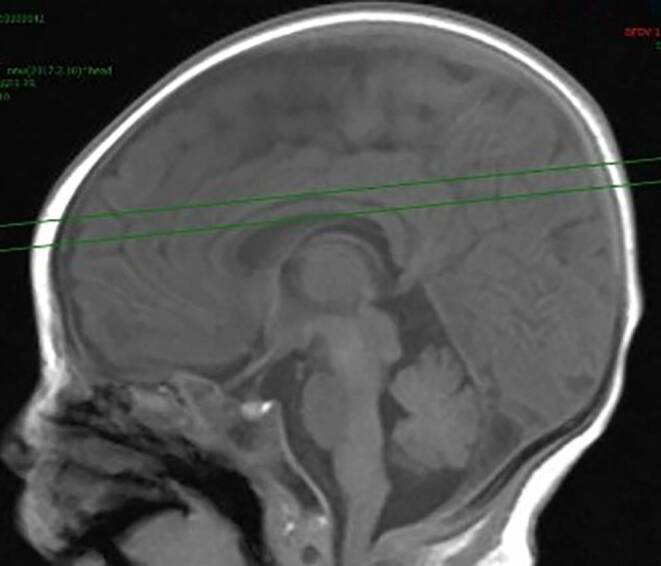
Positioning Map of reconstructed axial images used for quantitative analysis

**Fig. 2:**
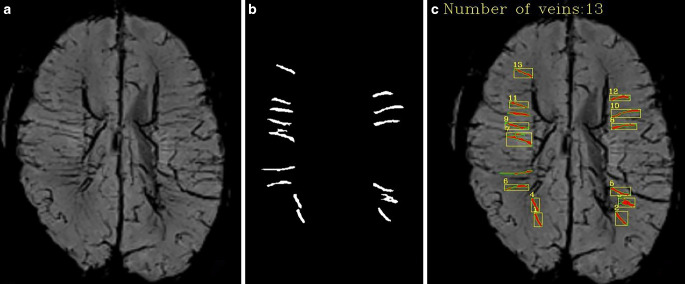
Automatic identification and quantitative analysis of DMVs. **a** Original MinIP image. **b** Divided DMVs. **c** Overlay and quantization of the segmented DMVs with the Original MinIP image

The cranial SWI images of 317 newborns enrolled in this study were processed with the same reconstruction parameters. The axial MinIP image data parallel to the body of corpus callosum and between its upper and lower edges (Fig. 1) were imported into the above model. The vessel diameter, curvature, and number of DMVs in bilateral cerebral hemisphere were automatically obtained (Fig. 2). The quantitative data were output in a text file, including the mean values of vessel diameter, vessel curvature, and number of DMVs displayed on the layer. The quantitative data of DMVs from each subject were organized for statistical analysis.

#### Data Collection of Thalamus Vein (TSV), Caudate Nucleus Antagonist Vein (ACV), and Intracranial Hemorrhage (ICH)

The localization of Bilateral Thalamus Vein (TSV) and Caudate Nucleus antagonist Vein (ACV) on the SWI MinIP map is based on the reports of Chen [21]. Using the Micro-DICOM viewer, the maximum diameter of the bilateral TSV and ACV were measured manually on the SWI MinIP image at the level of maximum display of the corresponding vessels (magnification 8×, WW/WL = 240/200). If more than one of the corresponding vessels was visible, the widest one was selected for measurement by repeating three times. The average value was recorded. If TSV or ACV were not detected in the SWI MinIP image, they were not included in the statistical analysis.

The number of newborns with intracranial extracerebral haemorrhage (ICECH) and intracranial intracerebral haemorrhage (ICICH) was recorded on T1WI and SWI sequences, respectively. ICECH included subarachnoid haemorrhage (SAH), subdural haemorrhage (SDH), and epidural haemorrhage (EDH). ICICH included periventricular-intraventricular haemorrhage (PVH-IVH), cerebellar haemorrhage (CH), and intraparenchymal haemorrhage (IPH).

The diagnostic criteria for ICH refer to the reports of Niwa [22] and Zhu [23] was as follows. (1) High signal and slightly high signal on T1WI were diagnosed as haemorrhagic foci after excluding vascular shadowing. (2) The low signal focus after excluding vascular shadowing on the SWI MinIP image, meanwhile mixed signals focus of high and low on the phase map were considered as haemorrhagic lesions. Foci with regular and difficult to confirm lesions at the skull base and under the skull were not included in the statistics.

### Statistical Analysis

SPSS 25.0 software was used for statistical analysis. The collected data was expressed as mean ± standard deviation ($\overline{\mathrm{x}}$ ± SD). The width of bilateral TSV and ACV, the number, the width and the curvature of DMV were all in skewed distribution. Therefore, the Mann-Whitney U test was used for the comparison between groups, the spearman correlation test was used for correlation analysis. *P* < 0.05 was considered significant.

## Results

### Quantitative Parameters of DMV in Bilateral Corona Radiata Area

The image segmentation algorithm model automatically identified and quantified DMV on the SWI MinIP images in 317 cases (Fig. 2a–c). The average number of identified DMV per case was 12.46 ± 5.58 (1–31), with a width of 0.73 ± 0.07 mm (0.49–0.93 mm) and a curvature of 1.10 ± 0.06 (range 1.03–1.57).

For all newborns, a comparison of quantitative parameters of the DMV between the relevant factor groups was shown in Table 1. The number of the DMV was more in males than in females (*P* < 0.01) and in term infants than in preterm infants (*P* = 0.001). The mean width of the DMV was greater in term than in preterm infants (*P* < 0.01), in low-risk than in high-risk groups (*P* < 0.01), and in children without ICECH than in those with ICECH (*P* < 0.05). The mean curvature of the DMV was greater in term than in preterm infants (*P* < 0.05).

**Table 1:** Intra-group differences of quantitative parameters of DMV in related factors ($\overline{\mathrm{x}}$ ± SD)

Items	Variables	The number of DMV	Z/*P*	The mean width of DMV	Z/*P*	The mean curvature of DMV	Z/*P*
Gender	Male	13.32 ± 6.00	Z = −2.638	0.73 ± 0.07	Z = −1.364	1.10 ± 0.06	Z = −0.521
	Female	11.50 ± 4.91	*P* = 0.008^**^	0.72 ± 0.07	*P* = 0.173	1.10 ± 0.07	*P* = 0.602
Term or not	Preter‑m	11.04 ± 4.43	Z = −3.232	0.70 ± 0.07	Z = −4.040	1.09 ± 0.07	Z = −2.521
	Term	13.23 ± 5.52	*P* = 0.001^**^	0.74 ± 0.07	*P* = 0.000^**^	1.10 ± 0.06	*P* = 0.012^*^
Delivery route	Cesarean	11.79 ± 5.75	Z = −1.791	0.72 ± 0.07	Z = −1.747	1.09 ± 0.05	Z = −0.887
	Vaginal	12.92 ± 5.43	*P* = 0.073	0.73 ± 0.07	*P* = 0.081	1.10 ± 0.07	*P* = 0.375
Risk group	High risk	12.29 ± 5.79	Z = −1.049	0.72 ± 0.07	Z = −2.686	1.10 ± 0.07	Z = −0.628
	Low risk	13.10 ± 4.72	*P* = 0.294	0.75 ± 0.07	*P* = 0.004^**^	1.10 ± 0.05	*P* = 0.530
NICH	YES	12.82 ± 5.67	Z = −1.417	0.72 ± 0.07	Z = −1.171	1.10 ± 0.06	Z = −0.275
	No	11.95 ± 5.43	*P* = 0.157	0.73 ± 0.08	*P* = 0.242	1.10 ± 0.07	*P* = 0.783
PVH-IVH	Yes	11.49 ± 5.89	Z = −0.852	0.72 ± 0.07	Z = −0.009	1.11 ± 0.07	Z = −0.386
	No	12.58 ± 5.54	*P* = 0.394	0.73 ± 0.07	*P* = 0.993	1.09 ± 1.62	*P* = 0.700
ICECH	Yes	12.45 ± 5.46	Z = −0.238	0.72 ± 0.07	Z = −2.140	1.09 ± 0.06	Z = −0.687
	No	12.48 ± 5.74	*P* = 0.812	0.73 ± 0.08	*P* = 0.032^*^	1.10 ± 0.07	*P* = 0.492

*Note*: Mann-Whitney U test, ^*^P < 0.05,^**^P < 0.01

*DMV* Deep medullary vein, *NICH* Neonatal intracranial haemorrhage, *PVH-IVH* Periventricular intraventricular haemorrhage, *ICECH* Intracranial extracerebral haemorrhage

In the high-risk group, the number (*P* < 0.01), length (*P* < 0.01) and curvature (*P* < 0.05) of DMV in full-term infants were also greater than those in premature infants (Table 2).

**Table 2:** Intra-group differences of quantitative parameters of DMV in high-risk group ($\overline{\mathrm{x}}$ ± SD)

Quantitative indicators of DMVs	Group (*n*)	Value	Z/*P*
The number of DMV	Term (138)	13.30 ± 5.89	Z = −2.983
	Preterm (111)	11.04 ± 5.43	*P* = 0.003^**^
The mean width of DMV	Term (138)	0.73 ± 0.072	Z = −3.173
	Preterm (111)	0.70 ± 0.074	*P* = 0.002^**^
The mean curvature of DMV	Term (138)	1.10 ± 0.063	Z = −2.417
	Preterm (111)	1.09 ± 0.069	*P* = 0.016^*^

*Note*: Mann-Whitney U test, ^*^P < 0.05,^**^P < 0.01

### Data Analysis of Bilateral TSVs and ACVs

Among 317 subjects, the rate of visualization of TSV in right and left cerebral hemisphere was 97.8% (310/317) and 98.6% (311/317) respectively. These TSV converged into the ipsilateral internal cerebral vein via the posterior border of the interventricular foramen and traveled mainly between the caudate nucleus and the thalamus. The rate of visualization of ACV was 96.8% (307/317) in both Cerebral hemisphere (Table 3).

**Table 3:** Occurrence rate (*n*, %) and width (mm, $\overline{\mathrm{x}}$ ± SD) of bilateral TSV and ACV

Venous name	Occurrence rate	Width
Right TSV	310(97.8)	1.59 ± 0.35(0.93–3.15)
Left TSV	311(98.6)	1.63 ± 0.38(0.79–2.91)
Right ACV	307(96.8)	1.23 ± 0.19(0.75–1.92)
Left ACV	307(96.8)	1.23 ± 0.21(0.72–2.22)

*Note*: Mann-Whitney U test, *P* > 0.05

*TSV* Thalamostriate vein; *ACV* anterior caudate vein

**Fig. 3:**
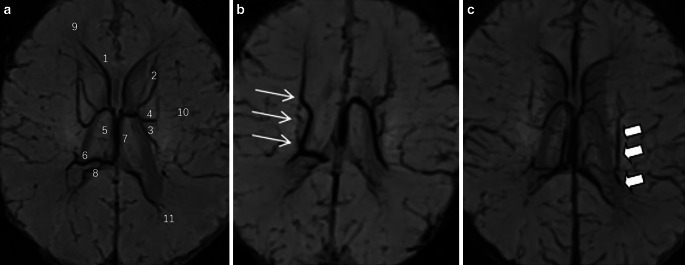
**a** Common routes of the TSV and ACV. 1. Anterior septal vein (ASV); 2. Anterior caudate vein (ACV); 3. Thalamostriate vein (TSV); 4. Transverse vein of caudate nucleus; 5 external straight vein; 6. Medial lateral ventricular vein (MLVV); 7. Internal cerebral vein (ICV); 8. Intraoccipital vein; 9. The deep medullary vein (DMV) flowing into ASV; 10. DMV flowing into transverse vein of caudate nucleus; 11. DMV flowing into MLVV. **b** The right TSV and the ACV are not shown, and an abnormal vein compensatory widening (thin white arrow) is seen locally. **c** The left TSV and ACV are thin, and an abnormally widened vein (thick white arrow) is seen locally

There were multiple variations in the location of the ACV into the intracerebral venous system, including separate or simultaneous convergence into the septal vein, the TSV, and the intersection of the septal and TSV (Fig. 3). Figure 3a shows the common course of the TSV and the ACV (Fig. 3a). Some of them may not be visualized, in which other anomalous veins with compensatory enlargement were often seen ipsilaterally to drain venous blood from the corresponding areas (Fig. 3b). In some patients, bilateral ACV and TSV were clearly observed with slim veins on one side, an abnormally widened vein could be seen nearby to drain blood to the medial vein of the lateral ventricles or the basal vein (Fig. 3c).

There were no statistically significant differences in the width of the bilateral TSV and ACV between the groups of sex, different term, delivery route, risk classification, presence of ICH, and presence of PVH-IVH (Table 4 and 5).

**Table 4:** Inter-group differences in the related factors of the width of bilateral TSVs (($\overline{\mathrm{x}}$ ± SD))

Items	Variables	R‑TSVs	Z/*P*	L‑TSVs	Z/*P*
Gender	Male	1.58 ± 0.36	Z = −1.180	1.63 ± 0.38	Z = −0.269
	Female	1.61 ± 0.34	*P* = 0.238	1.63 ± 0.38	*P* = 0.788
Term or not	Preterm	1.57 ± 0.38	Z = −1.311	1.65 ± 0.39	Z = −0.753
	Term	1.60 ± 0.33	*P* = 0.190	1.62 ± 0.37	*P* = 0.451
Delivery route	Cesarean	1.56 ± 0.29	Z = −1.085	1.61 ± 0.34	Z = −0.002
	Vaginal	1.62 ± 0.38	*P* = 0.278	1.64 ± 0.40	*P* = 0.998
Risk group	High risk	1.59 ± 0.35	Z = −0.298	1.62 ± 0.38	Z = −1.012
	Low risk	1.61 ± 0.37	*P* = 0.766	1.66 ± 0.38	*P* = 0.312
NICH	Yes	1.61 ± 0.35	Z = −1.348	1.62 ± 0.38	Z = −0.304
	No	1.58 ± 0.35	*P* = 0.178	1.64 ± 0.37	*P* = 0.761
PVH-IVH	Yes	1.65 ± 0.43	Z = −0.348	1.72 ± 0.44	Z = −1.001
	No	1.59 ± 0.34	*P* = 0.727	1.62 ± 0.37	*P* = 0.317

*Note*: Mann-Whitney U test, ^*^P < 0.05, ^**^P < 0.01

*TSV* Thalamostriate vein, *NICH* Neonatal intracranial haemorrhage, *PVH-IVH* Periventricular intraventricular haemorrhage

**Table 5:** Inter-group differences in the related factors of the width of bilateral ACV (($\overline{\mathrm{x}}$ ± SD))

Items	Variables	R‑ACV	Z/*P*	L‑ACV	Z/*P*
Gender	Male	1.24 ± 0.20	Z = −1.430	1.24 ± 0.21	Z = −1.125
	Female	1.21 ± 0.18	*P* = 0.153	1.21 ± 0.21	*P* = 0.260
Term or not	Preterm	1.24 ± 0.19	Z = −0.869	1.24 ± 0.21	Z = −0.867
	Term	1.22 ± 0.19	*P* = 0.385	1.22 ± 0.21	*P* = 0.386
Delivery	Cesarean	1.22 ± 0.17	Z = −0.427	1.22 ± 0.18	Z = −0.267
	Vaginal	1.23 ± 0.21	*P* = 0.669	1.23 ± 0.23	*P* = 0.789
Risk group	High risk	1.23 ± 0.19	Z = −0.917	1.23 ± 0.21	Z = −0.322
	Low risk	1.21 ± 0.21	*P* = 0.359	1.22 ± 0.19	*P* = 0.749
NICH	Yes	1.22 ± 0.19	Z = −1.493	1.21 ± 0.21	Z = −1.496
	No	1.24 ± 0.19	*P* = 0.135	1.25 ± 0.21	*P* = 0.135
PVH-IVH	Yes	1.24 ± 0.19	Z = −0.093	1.23 ± 0.24	Z = −0.083
	No	1.23 ± 0.19	*P* = 0.926	1.23 ± 0.20	*P* = 0.934

*Note*: Mann-Whitney U test, *P < 0.05, ^**^P < 0.01

*ACV* anterior caudate vein, *NICH* Neonatal intracranial haemorrhage, *PVH-IVH* Periventricular intraventricular haemorrhage

Spearman correlation analysis between the width of bilateral TSV & ACV and the parameters of DMV in the drainage area is shown in Table 6. The width of the bilateral TSV and ACV were positively correlated with the number of the DMV. The width of the bilateral TSV was positively correlated with the mean width of the DMV.

**Table 6:** Correlation analysis of parameters of bilateral TSV, ACVs and DMV

	Number of DMV	Average width of DMV	Mean curvature of DMV
R‑TSVs	Rs = 0.238, *p* = 0.000^**^	Rs = 0.195, *p* = 0.001^**^	Rs = 0.104, *p* = 0.068
L‑TSVs	Rs = 0.271, *p* = 0.000^**^	Rs = 0.257, *p* = 0.000^**^	Rs = 0.093, *p* = 0.100
R‑ACVs	Rs = 0.205, *p* = 0.000^**^	Rs = 0.110, *p* = 0.054	Rs = 0.040, *p* = 0.488
L‑ACVs	Rs = 0.230, *p* = 0.000^**^	Rs = 0.099, *p* = 0.083	Rs = 0.112, *p* = 0.051

*Note*: Spearman rank correlation analysis, ^*^P < 0.05, ^**^P < 0.01

*DMV* Deep medullary vein, *TSV* Thalamostriate vein, *ACV* anterior caudate vein

## Discussion

### The Clinical Significance of Quantitative Parameters of Bilateral DMVs in Vivo

The medullary veins, first named by Duret in 1874 [24], include the deep and superficial medullary veins, which are mainly located in the white matter of the cerebral and cerebellar hemispheres and communicate with the deep intracranial venous system and the superficial venous system. The superficial medullary vein drains the white matter 1–2 mm below the cortex, while the DMV drains the remaining deep white matter venous blood. The supratentorial DMVs are arranged in wedge-shaped patterns [24]. In the anterior hemisphere, during the drainage of DMVs to the upper lateral corner of the frontal horn of the lateral ventricle, four venous convergence areas are formed. DMVs in the deep white matter of the superior frontal gyrus drain into transparent septal vein, while DMVs in the deep white matter of the inferior frontal gyrus drain into ACV. In the middle hemisphere, DMVs located adjacent to the body of the lateral ventricles course perpendicular to the lateral ventricles, drain into the transverse vein of the caudate nucleus, and then entering TSV. In the posterior hemisphere, DMVs distributed radially along the occipital horn of the lateral ventricle drain into MLVV [21, 24–26].

In 2008, Tong et al. observed that the number and width of medullary veins in deep white matter lesions increased on SWI images, and named as DMV signs [26]. In children with acute ischemic stroke, more DMV can also be observed [27, 28] on SWI images. The possible pathophysiological mechanisms include [4, 29]: (1) The opening of collateral circulation in brain tissue regions with reduced perfusion lead to secondary dilation of corresponding drainage venules. (2) Local ischemia- hypoxia cause an increase of deoxyhaemoglobin in the vein vessels, making the veins on the SWI images more easily visible.

The pathophysiological mechanisms of neonatal hypoxic-ischemic encephalopathy (HIE) are complex, and the pathophysiological changes that occur at different stages of the disease also vary [12, 16, 19, 30, 31]. The main pathological changes include abnormal brain perfusion due to hypoxia-ischemia, brain cell edema, selective neuronal necrosis, cerebrovascular autoregulation dysfunction, and cerebral infarction. It is often secondary to cerebral venous stasis and dilation. Due to the immature vascular development, the basement membrane of cerebral vein is easy to be damaged and lead to venous leakage of haemoglobin & red blood cells and venous infarction [12, 16, 19, 30, 31].

Abnormal oxygenation and/or perfusion changes usually precede brain cell injury [19, 32, 33]. Treatment targeting one or more stages of pathophysiology has been proven to significantly improve disability free survival for HIE neonates [16]. Some studies have confirmed that the DMV in neonates with moderate-to-severe neonatal HIE is significantly more dilated than in normal neonates [34]. Therefore, DMV can be used as potential indicators for observing the pathophysiology of cerebral circulatory compensation in various stages of HIE, diagnosing the degree of brain injury, and evaluating its prognosis in vivo.

Kitamura et al. proposed the evaluation of DMV display level based on MinIP image of SWI sequence, and developed a DMV display level system [18]. Niu Gang et al. proposed a quantitative method for identifying DMV through threshold quantitative segmentation on SWI MinIP image and calculating the ratio of corresponding vascular area to total area of interest (vein-ROI ratio, VSR), which has some promise for use in quantitative studies to evaluate the degree of hypoxia in HIE [35].

In this study, an image segmentation algorithm was used to automatically identify the DMV of bilateral corona radiata by software and calculate the width and curvature data of each vessel. After training, computer algorithms can obtain relevant data based on objective and uniform criteria, with high repeatability and higher efficiency than manual measurements. DMV are slender and have a large number of blood vessels, resulting in significant errors and difficulty in ensuring measurement accuracy by manual measurement. Compared to the evaluation method proposed by Kitamura et al., this method can provide objective quantitative data with high efficiency.

The results of this study showed that the mean number (*P* < 0.01), mean width (*P* < 0.01), and mean curvature (*P* < 0.05) of DMV in term infants with different hypoxic-ischemic risk factors were greater than those in preterm infants. This trend is consistent with the compensatory mechanism of cerebral blood flow in the early stage of neonatal hypoxic-ischemia. In the early stage of hypoxic ischemia in brain tissue, the body initiates the compensatory mechanism to reduce the blood flow supply of other organs, and the cerebral blood flow can increase to 2–3 times of normal [36] to ensure the energy supply of brain tissue. At this time, it can be seen that the number and diameter of corresponding medullary veins increases. The vascular regulation against hypoxia-ischemia is better in full-term infants than in preterm infants [19]. The results of the present study confirm in vivo that term infants have more mature cerebrovascular development than preterm infants, and their vascular regulation is more compensatory than that of preterm infants.

However, the number of DMV in males was greater than that in females (*P* < 0.01). It is speculated that after the occurrence of cerebral tissue hypoxia-ischemia, the vascular regulation and compensation ability of males is stronger than that of females. At this time, there is an increase in cerebral blood flow and venous reflux, and an increase in the number of venous vessels that can be displayed. Because of the imperfect vascular development for neonates, the vein basement membrane damage is likely to occur, causing leakage of haemoglobin and red blood cells from the stagnant dilated veins, and the risk of venous rupture and bleeding is correspondingly increased. Our previous analysis of ICH in neonates also suggested that the incidence of ICECH was higher in males than in females (*P* < 0.05) [37].

Statistical analysis of our cases suggested that the mean width of DMV in children in the high-risk group of hypoxia-ischemia was less than that in the low-risk group (*P* < 0.01), which is not consistent with previous literature that reported widening of the DMV in children with neonatal HIE [34]. The possible reason for this is that children with a high risk of ischemia-hypoxia are not equivalent to children with neonatal HIE. In addition, premature infants accounted for 44.58% (111/249) of high-risk newborns in this study, which may be the reason for the results of this study. From the perspective of whether the vascular regulation function is activated or not, the results of this study can also reflect the early stage of hypoxic-ischemic factors. Before the activation of the vascular regulation function, due to the reduction of cerebral blood flow perfusion, the corresponding venous reflux is reduced, and the diameter of the DMV is smaller than that of the newborn without hypoxic-ischemic factors. According to literature report [34], there was no statistically significant difference in the width of the medullary vein between the control group and mild HIE, only children with moderate to severe HIE had greater width of the medullary vein than that of children in control group.

In addition, the width of the DMV in children with neonatal ICECH is smaller than that in children without neonatal ICECH (*P* = 0.032). It may reflect that cerebral blood flow in neonates with ICECH is reduced by vascular autoregulation function to reduce the volume of venous blood and DMV vasoconstriction, which is self-rescue to reduce blood extravasation, so as to avoid continuous bleeding.

### TSV, ACV and Observation of the DMVs Reflux in Vivo

The three pairs of main branches of internal cerebral vein (ICV) include bilateral anterior septal vein (ASV), TSV and medial lateral ventricular vein (MLVV), which drain venous blood from the DMV in the lateral paraventricular area [21, 25]. Because ASV and MLVV mainly drain blood from the DMV in the area adjacent to the anterior and posterior horn of the lateral ventricle, which are not in the drainage area of DMV observed in this study. The bilateral paraventricular middle DMV drain mainly to the bilateral TSV and another secondary branch of the internal cerebral veins—ACV [21].

The TSV travels mostly between the caudate nucleus and the thalamus. The variations of TSV include two TSV on one side, or a slender main trunk accompanied by compensatory widening of adjacent veins. The location, number and width of bilateral TSV can be asymmetrical [38]. Most of the bilateral ACV are accompanied by multiple branches, and their confluence into ICV is diverse, which can respectively or simultaneously join ASV, TSV and the angle of TSV and ASV [38]. In a study of 40 adult volunteers [39], 70 ACV were shown bilaterally, mainly joining the ipsilateral TSV in 64.3% (45 cases), joining ASV in 14.3% (10 cases), joining the angle of TSV and ASV in 21.4% (15 cases).

The bilateral TSV and ACV drained venous blood from the deep white matter of the lateral paraventricular and basal ganglia nuclei and thalamus to ICV. The corresponding veins of neonates with HIE often have abnormal changes [18]. When neonatal PVH-IVH, periventricular leukomalacia as well as damage of neurons in basal ganglia gray matter nucleus, thalamus and brainstem occur, congestion and dilation of DMV and subependymal veins at corresponding sites are often seen [8]. In this study, Spearman rank correlation analysis was performed between the width of bilateral TSV & ACV and the parameters of corresponding DMV. The results showed that the width of bilateral TSV and ACV was positively correlated with the number of corresponding DMV, the width of bilateral TSV was positively correlated with the width of DMV. It can be confirmed by MR in vivo that the blood drained from the corresponding deep cerebral white matter by bilateral DMV mainly flows into ICV through bilateral TSV. The width of the corresponding DMV was only positively correlated with the width of the TSV, but not with ACV. Possible reasons for this include: (1) Only a small part blood from bilateral paraventricular middle DMV flows to ACV, and more blood flows back to ICV through TSV [21]. (2) ACV is often multibranched, and when the corresponding DMV blood flow changes, the width of a single ACV vessel does not change significantly due to multiple branch shunts. (3) There may be widened variant vessels near the ACV, which drain blood to MLVV or basal vein, resulting in a decrease in blood flow through the ACV. Therefore, the change of DMV blood flow is difficult to cause the change of the width of the ACV.

### Limitations

First, the quantitative study of the DMV requires training of the image segmentation algorithm using a large amount of data, but the pre-learning samples are relatively small. The training of the algorithm relies on the corresponding vascular images manually depicted by the pre-reading radiologist, which makes it difficult to control the quality of the learning samples. The further studies are needed to increase the learning samples and further optimize the algorithm. Second, this study was aimed at infants who suffered from hypoxic-ischemic risk factors and lack of neonates who had moderate-severe neonatal HIE, so the application value of quantitative study of DMV in neonatal moderate-severe HIE needs further exploration. Third, the MinIP image of SWI has amplification effect. In addition, the intensity of venous signal is closely related to the content of deoxyhaemoglobin (paramagnetic substances) in vein vessels. Therefore, vein vessels with the same diameter may show differences in width due to different content of deoxyhaemoglobin in the venous blood [4]. Similarly, the measured value of medullary vein based on SWI MinIP image may differ from the actual value of vascular anatomy. We do not have data on blood oxygen saturation during neonatal MR examination and have not conducted relevant comparative studies, which is also a limitation of this study.

However, computerized image segmentation algorithms can repeat multiple measurements with objective and uniform criteria while maintaining consistent results, giving a promising method for quantification of intracranial veins. It has tremendous advantages over subjective visual judgments and manual measurements, and can reflect abnormal changes of the medullary vein in the pathological state, providing us with more, stable and meaningful information.

## Conclusions

The results of this study may suggest that DMV measurement method based on image segmentation algorithm can provide more detailed and relatively stable quantitative vascular data, which can reflect the venous vascular alterations in neonatal brain caused by cerebral hypoxic-ischemic factors. The positive correlation between the width of bilateral TSV and the number and width of bilateral DMV confirms that the change in the width of bilateral TSV can indirectly reflect the hemodynamic changes of the corresponding DMV, which may be an in vivo observation indicator of neonate blood circulation self-regulation under the influence of neonatal hypoxic-ischemic risk factors.

## Data Availability

Study data are available upon reasonable request to the corresponding authors, and after clearance by the local ethics committee.

## Abbreviations

ACV: Caudate Nucleus Anterior Vein
CH: Cerebellar Haemorrhage
DMV: Deep Medullary Vein
EDH: Epidural Haemorrhage
FOV: Field of View
ICECH: Intracranial Extracerebral Haemorrhage
ICH: Intracranial Haemorrhage
ICICH: Intracranial Intracerebral Haemorrhage
IPH: Intraparenchymal Haemorrhage
MinIP: Minimum Intensity Projection
MRI: Magnetic Resonance Imaging
PVH-IVH: Periventricular-Intraventricular Haemorrhage
SAH: Subarachnoid Haemorrhage
SDH: Subdural Haemorrhage
SWI: Susceptibility Weighted Imaging
TE: Echo Time
TR: Repetition Time
TSV: Thalamus Vein
T1WI: T1 Weighted Imaging
WMI: White Matter Injury
